# The relationships of both transition shock, empathy, resilience and coping strategies with professional quality of life in newly graduated nurses

**DOI:** 10.1186/s12912-021-00589-0

**Published:** 2021-04-23

**Authors:** Xiaoyi Cao, Jin Li, Shu Gong

**Affiliations:** Department of Nursing, West China Hospital/West China School of Nursing, Sichuan University, 37, Guoxue Road, Chengdu, Sichuan Province People’s Republic of China

**Keywords:** Compassion fatigue, Compassion satisfaction, Coping strategies, Empathy, Newly graduated nurses, Professional quality of life, Resilience, Transition shock

## Abstract

**Background:**

Data on professional quality of life in newly graduated nurses are scarce. This study aimed to describe the levels of professional quality of life, and to explore the relationships of transition shock, empathy, resilience and coping strategies with professional quality of life in newly graduated nurses.

**Methods:**

This was a cross-sectional study, which used a two-stage sampling method to recruit 393 newly graduated nurses in Sichuan province of China. Multiple regression analysis was used to explore the effects of transition shock, empathy, resilience and coping strategies on professional quality of life. Data were collected using standardized scales.

**Results:**

The prevalence of average levels of compassion satisfaction, burnout and secondary traumatic stress in newly graduated nurses were 80.2, 38.2 and 57.5%, respectively. Transition shock was a significant negative predictor, and empathy, resilience and adaptive coping were significant positive predictors for compassion satisfaction. Transition shock and passive coping were significant positive predictors, and empathy was a significant negative predictor for burnout and secondary traumatic stress. Resilience and adaptive coping contributed to burnout significantly and negatively.

**Conclusion:**

Higher transition shock and lower empathy cause lower compassion satisfaction and higher compassion fatigue. More resilience and adaptive coping cause more compassion satisfaction and less burnout. More passive coping contributes to higher compassion fatigue. Strategies such as transition or preceptorship programmes, and empathy, resilience and coping training are effective methods to reduce transition shock, facilitate empathy, resilience and coping, and consequently, enhance professional quality of life in newly graduated nurses.

## Background

The nursing shortage receives global attention, which affects the quality of care. According to the latest data, the number of nurses worldwide is 20.7 million in 2013, and another 11.6 million nurses will be required in 2030 [1]. In China, although 4.1 million nurses are employed in 2018 [2], the nurses per 1000 inhabitants are 2.7, which are drastically lower than those in Western countries [3]. Therefore, it is essential to establish strategies for retaining experienced nurses and attracting newly graduated nurses (NGNs) to alleviate the ongoing nursing workplace crisis.

Professional quality of life represents cumulative positive (compassion satisfaction, CS) and negative experience (compassion fatigue, CF) caregivers perceive regarding their work as professional helpers [4], which affects the health and effectiveness of nurses. Recently, professional quality of life in nurses received much concern, especially for those employed in the emergency rooms, intensive care units and oncology wards [5]. Previous studies discovered that, higher CS and lower CF significantly contributed to higher job satisfaction, and lower anxiety, depression and turnover intention in experienced nurses [6, 7]. As entry-level nurses, NGNs encountered great challenges in the transition to clinical practice, which resulted in high job stress, burnout and turnover intention [8]. Moreover, most existing studies explored the subjects of stress, job satisfaction, burnout and turnover, little attention was paid to professional quality of life in NGNs [6–8]. Thus, to examine the mechanism which affected professional quality of life in NGNs might contribute to developing strategies for facilitating CS, alleviating CF, and reducing turnover intention eventually.

Professional quality of life refers to the quality caregivers perceive as professional helpers, which incorporates CS and CF [4]. Of these, CS refers to the pleasure of a caregiver, which derives from being capable of engaging in work and dealing with costs of caring well. CF represents a caregiver’s diminished capacity to care due to repeated exposure to the suffering of clients, which breaks into two parts: burnout and secondary traumatic stress (STS). The first part is associated with negative feelings of frustration and exhaustion related to difficulties in handling work effectively. The second part is associated with negative feelings triggered by work-related, secondary exposure to clients who suffer from traumatic events [4]. Recently, professional quality of life in nurses received widespread attention, which affected turnover, retention and their ability to provide care. Previous studies uncovered that, CS was less prevalent and CF was more prevalent in nurses with less years of job experience [6, 9, 10]. Another study found that, nurses with less than 3 years of experience reported lower CS and higher CF [11]. However, most existing studies explored professional quality of life and its related factors in experienced nurses, fewer studies examined these variables in NGNs with less years of job experience, who might perceive less CS and more CF in their transition to clinical practice.

The professional quality of life model was used to guide this study. Based on this model, positive (CS) or negative (CF) aspects of helping others can be affected by demographic, job-related and personal factors. Of those, demographic variables included age, gender, years of job experience and educational level; job-related variables included job stress, and support from managers and peers; and personal variables comprised personality, empathy and resilience [4]. Thus, according to the model, the following hypotheses were formulated: 1) as a job-related factor, transition shock resulting from poor adaptation to clinical practice was negatively and significantly associated with CS, and positively and significantly associated with CF in NGNs; and 2) as personal resources, empathy, resilience and coping strategies were significantly and positively associated with CS, and significantly and negatively associated with CF, respectively.

NGNs usually experience considerable emotional burden during their transition to clinical practice [12]. To date, most existing studies focused on the description of transition experience in NGNs using qualitative studies. Meanwhile, some quantitative studies discovered a high prevalence of transition shock in NGNs despite differences in severity, and transition shock was a significant predictor for nurse turnover, job involvement, negative emotional states and adverse patient events [13]. However, no studies examined the relationship between transition shock and professional quality of life in this population.

Interestingly, regarding the association between empathy and professional quality of life in nurses, it was found that, empathy was the main etiology for CF [14]. Empathy was positively and significantly related to CS, but negatively and significantly related to CF among nurses in China and Portugal [15, 16]. Furthermore, empathy was a significant contributor to CS in oncology nurses [17]. Therefore, although the significant associations between empathy and professional quality of life in experienced nurses were demonstrated across prior studies, no studies examined the correlation between empathy and professional quality of life in NGNs.

Resilience was a significant contributor to nurse turnover intention and work performance [18]. With regard to the relationship between resilience and professional quality of life in nurses, it was uncovered that, resilience was positively and significantly related to CS, but negatively and significantly related to CF in Australian nurses [19]. Higher resilience contributed to higher CS and lower CF significantly in Australian critical care nurses [20]. Accordingly, although there were consistencies in the associations between resilience and professional quality of life in experience nurses, little attention was paid to the relationships between these variables in NGNs.

Moreover, coping strategies affect nurse burnout, job satisfaction, turnover and retention. Regarding the associations between coping strategies and professional quality of life, a previous study on cancer nurses found that, passive coping was positively and significantly related to CF, but adaptive coping was not positively and significantly related to CS [17]. Another study on Australian critical care nurses revealed that, coping strategies had no significant effects on CS and CF, respectively [20]. Moreover, problem-solving coping significantly predicted CS, and avoidance coping significantly predicted CF among critical care nurses in Jordan [21]. Thus, inconsistent results existed in the correlations between coping strategies and professional quality of life in experienced nurses. Meanwhile, no studies explored whether and to which extent coping strategies affected professional quality of life in NGNs.

The aims of the study were to describe the levels of professional quality of life, and to examine the relationships of transition shock, empathy, resilience and coping strategies with professional quality of life in Chinese NGNs.

## Methods

### Study design and setting

This was a cross-sectional study conducted in Sichuan province, which is located in the southwest region of China. The formula of sample size calculation used in this study was [*n* = (u_α_σ/δ)^2^]. As no prior studies reported CS or CF in NGNs, the calculation of sample size was based on the scores of CS in the pilot study (mean = 37.69, standard deviation = 5.02). A minimal sample size of 387 was required based on the expected error of estimation of 0.5 and a 5% margin of error.

A two-stage sampling method was used. First, 10 tertiary hospitals in Sichuan province were selected (2 tertiary hospitals were chosen from eastern, southern, western, northern and middle regions of Sichuan province, respectively). Then, all of the NGNs in the selected hospitals were recruited. The NGNs meeting the following criteria were included: 1) having a nursing license; and 2) having job experience of no more than 1 year. The NGNs with maternity leave or sick leave during the survey were excluded. Finally, of 475 participants eligible, 393 participants voluntarily and successfully completed the survey with a response rate of 82.7%.

### The study questionnaire

Transition shock scale for newly graduated nurses assesses emotional reactions (e.g., doubt, confusion and disorientation) in NGNs due to the conflicting theories and realities in clinical practice. It comprises 33 items and 4 domains (physical shock, psychological shock, shock from knowledge and skills, and shock from organizational culture and climate). Each item is rated by a 5-point Likert scale with higher scores indicating higher transition shock. The original Chinese scale indicates a satisfactory structural validity, and the Cronbach’s α of each subscale ranges from 0.86 to 0.94 [22]. In this study, the Cronbach’s α of each subscale ranged from 0.81 to 0.89.

Jefferson Scale of Empathy appraises empathic qualities in providing care, which comprises 20 items and 3 subscales (standing in the patient’s shoes, compassionate care and perspective taking). It is ranked by a 7-point Likert scale with higher scores indicating higher empathic ability. Exploratory factor analysis indicates a satisfactory structural validity, and the overall scale has a Cronbach’s α of 0.80 [23]. The Chinese version also has acceptable psychometric properties [24]. In this study, the Cronbach’s α of each subscale ranged from 0.60 to 0.82.

Connor-Davidson Resilience Scale appraises the ability to deal with adversity. It is a 10-item instrument with an uni-dimensional structure. It is rated by a 5-point Likert scale with lower scores indicating lower resilience. Exploratory factor analysis supports a one-factor construct and the Cronbach’s α of the scale is 0.85 [25]. The Chinese scale also indicates an excellent internal consistency, test-retest reliability and structural validity [26]. The Cronbach’s α of the scale in this study was 0.92.

Simple Coping Style Questionnaire evaluates coping strategies to handle challenges under adversity. It is a 20-item instrument with 2 domains (adaptive coping and passive coping). The Chinese questionnaire is scored on a 4-point Likert scale, and each subscale is rated independently with higher scores indicating higher adaptive and passive coping. The Cronbach’s α of the overall scale is 0.90 [27]. In this study, the Cronbach’s α of each subscale ranged from 0.78 to 0.87.

Professional Quality of Life Scale assesses positive and negative aspects of helping others who suffer from traumatic events. It comprises 30 items and 3 domains (CS, burnout and STS), with the latter 2 domains reflecting CF. The scale is rated by a 5-point Likert scale, and each domain is ranked independently with higher scores indicating higher CS, burnout and STS. The score of each domain ranges from 10 to 50 and is classified into 3 levels (22 or less: low level, 23 to 41: average level, 42 or more: high level). Exploratory factor analysis supports a 3-factor construct, and the Cronbach’s α of each domain ranges from 0.72 to 0.87 [4]. The Chinese version also has satisfactory psychometric properties [28]. In this study, the Cronbach’s α of each domain ranged from 0.78 to 0.91.

### Data collection

Prior to the study, permission was obtained from each president of the selected hospitals. Then, one nurse with a Bachelor degree in nursing science from each hospital was selected as a research assistant to participate in a 2-h online training session, which comprised the objectives of the study, the inclusion and exclusion criteria of the participants, and the process of data collection. The data was collected via an online survey, and a QR code was designated and distributed to each research assistant before the survey. The procedure of data collection was as follows: first, based on the inclusion and exclusion criteria, the research assistant in each hospital chose potential participants and introduced the aims of the study in a meeting room during day shift. Next, the potential participants were informed that participation was voluntary and anonymous, they were required to scan a designated QR code using their own smartphones, and an electronic written informed consent was required to be signed. Finally, the participants were instructed to complete and submit the online questionnaires. The study was conducted from Jul 1 to Aug 31, 2020.

### Statistical analysis

SPSS 23.0 was used for data analysis. As each subscale of professional quality of life approximated normality, the differences in each subscale between subgroups were examined using independent samples t-tests or one-way analysis of variances. Pearson correlation analysis was used to explore the relationships between these variables. The effects of demographic factors, transition shock, empathy, resilience and coping strategies on each subscale of professional quality of life were examined using multiple regression analysis. *P* < .05 was statistically significant (two-tailed test).

### Ethical approval

Ethical approval was obtained from the Human Subjects Ethics Sub-committee of West China Hospital of Sichuan University (no. 2020281). The informed consent was obtained from all of the subjects. The participants could participate in the study voluntarily and could withdraw when they wanted it at any time. All methods in this study were carried out in accordance with relevant guidelines and regulations.

## Results

### Sample characteristics

The respondents included 71 (18.1%) men and 322 (81.9%) women. The average age was 22.45 (standard deviation = 1.02) ranging from 20 to 25 years. Over half of the respondents had a bachelor degree or above (65.1%), and 58.3% of the respondents had siblings. Most of the respondents worked in a medical (29.3%) or surgical unit (24.7%), and experienced patient death (69.0%). Moreover, 30.3% of the respondents received empathy training (Table 1).

**Table 1:** Differences in each subscale of professional quality of life among subgroups (*n* = 393)

Variables	n (%)	CS mean ± standard deviation	Burnout mean ± standard deviation	STS mean ± standard deviation
Sex				
Male	71 (18.1)	37.83 ± 5.58	20.77 ± 5.22	22.11 ± 4.66
Female	322 (81.9)	37.87 ± 5.07	21.41 ± 5.12	23.51 ± 4.26
t (*p*)		− 0.052 (0.958)	− 0.948 (0.344)	− 2.459 (0.014)
Educational level				
Associate degree or below	137 (34.9)	38.88 ± 4.95	19.93 ± 4.88	22.59 ± 4.35
Bachelor degree or above	256 (65.1)	37.31 ± 5.19	22.03 ± 5.13	23.61 ± 4.33
t (*p*)		2.906 (0.004)	−3.918 (0.000)	−2.226 (0.027)
Having siblings				
No	164 (41.7)	37.73 ± 5.74	21.26 ± 5.22	23.20 ± 4.23
Yes	229 (58.3)	37.95 ± 4.70	21.33 ± 5.09	23.30 ± 4.45
t (*p*)		− 0.417 (0.677)	−0.136 (0.892)	− 0.214 (0.830)
Unit of assignment				
Medical unit	115 (29.3)	37.80 ± 4.90	21.17 ± 4.96	23.20 ± 4.24
Surgical unit	97 (24.7)	38.30 ± 5.78	20.84 ± 5.44	22.87 ± 4.19
Emergence care unit	14 (3.6)	37.79 ± 5.62	21.86 ± 5.87	23.29 ± 5.37
Intensive care unit	33 (8.4)	37.48 ± 4.56	22.52 ± 5.19	24.03 ± 4.48
Operating unit	63 (16.0)	37.10 ± 5.22	22.25 ± 5.33	24.19 ± 4.63
Other unit	71 (18.1)	38.23 ± 4.84	20.61 ± 4.57	22.69 ± 4.24
F (*p*)		0.525 (0.758)	1.273 (0.275)	1.188 (0.314)
Having experienced patient death				
Yes	271 (69.0)	37.84 ± 5.35	21.42 ± 5.27	23.38 ± 4.38
No	122 (31.0)	37.91 ± 4.72	21.02 ± 4.84	22.98 ± 4.32
t (*p*)		− 0.128 (0.898)	0.728 (0.467)	0.834 (0.405)
Empathy training				
Yes	162 (30.3)	38.78 ± 5.06	20.42 ± 5.03	22.81 ± 4.39
No	231 (69.7)	37.22 ± 5.14	21.91 ± 5.13	23.57 ± 4.32
t (*p*)		2.985 (0.003)	−2.863 (0.004)	−1.688 (0.042)

*CS* compassion satisfaction, *STS* secondary traumatic stress

### The levels of professional quality of life

The prevalence of average and high levels of CS were 80.2% (315 nurses) and 19.8% (78 nurses). A total of 243 NGNs (61.8%) reported low burnout, and 150 (38.2%) reported average burnout. Moreover, the prevalence of low and average levels of STS were 42.5% (167 nurses) and 57.5% (226 nurses).

The mean scores of CS, burnout and STS were 37.86 (standard deviation = 5.16), 21.30 (standard deviation = 5.14) and 23.56 (standard deviation = 4.36), respectively. NGNs with an associate degree or below reported significant higher CS, and significant lower burnout and STS than those with a bachelor degree or above. Compared to NGNs without empathy training, those receiving empathy training reported significant higher CS, and significant lower burnout and STS. Moreover, female nurses reported significant higher STS than male nurses, but no significant differences on CS and burnout were uncovered between female and male nurses. No significant differences on each subscale were found in NGNs with different units of assignment, with or without siblings, and with or without patient death experience (Table 1).

### Relationships of transition shock, empathy, resilience and coping strategies with professional quality of life

Transition shock and passive coping had significant negative relationships with CS, and significant positive relationships with burnout and STS. Empathy, resilience and adaptive coping were significantly and positively related to CS, and significantly and negatively related to burnout and STS, respectively (Table 2).

**Table 2:** Relationships of transition shock, empathy, resilience and coping strategies with professional quality of life (*n* = 393)

Variables	TS	Empathy	Resilience	AC	PC	CS	Burnout	STS
TS	1.000	−0.426***	−0.557***	− 0.290***	0.356***	− 0.556***	0.723***	0.631***
Empathy		1.000	0.600***	0.491***	−0.297***	0.618***	−0.593***	−0.370***
Resilience			1.000	0.558***	−0.292***	0.689***	−0.684***	−0.428***
AC				1.000	0.034	0.482***	−0.430***	−0.237***
PC					1.000	−0.294***	0.387***	0.362***
CS						1.000	−0.825***	− 0.403***
Burnout							1.000	0.669***
STS								1.000

*** *P* < 0.001. AC adaptive coping, *CS* compassion satisfaction, *PC* passive coping, *STS* secondary traumatic stress, *TS* transition shock

### Effects of demographic data, transition shock, empathy, resilience and coping strategies on professional quality of life

Based on the results of independent samples t-tests or one-way analysis of variances, significant demographic factors including sex, educational level and empathy training were incorporated into multiple regression analysis. The findings indicated that, transition shock, empathy, resilience and adaptive coping were significant contributors to CS, explaining 57.6% of the variance. Resilience was the strongest predictor (*β* = .343, *p* < .001), followed by empathy (*β* = .259, *p* < .001), transition shock (*β* = −.192, *p* < .001) and adaptive coping (*β* = .107, *p* < .05). Transition shock, empathy, resilience, and adaptive and passive coping were significant predictors for burnout, which explained 67.8% of the variance, with transition shock as the strongest contributor (*β* = .430, *p* < .001), followed by resilience (*β* = −.247, *p* < .001) and empathy (*β* = −.189, *p* < .001). NGNs with a bachelor degree or above reported significant higher burnout than those with an associate degree or below (*β* = .080, *p* < .01). Moreover, transition shock, empathy and passive coping predicted STS significantly, explaining 42.0% of the variance. Transition shock was the strongest contributor (*β* = .514, *p* < .001), followed by passive coping (*β* = .149, *p* < .01) and empathy (*β* = −.100, *p* < .05) (Table 3).

**Table 3:** Effects of demographic data, transition shock, empathy, resilience and coping strategies on professional quality of life (*n* = 393)

Variables	CS			Burnout			STS		
	β	t	p	β	t	p	β	t	p
Constant	–	6.044	0.000	–	12.627	0.000	–	5.832	0.000
Sex (male/female)	–	–	–	–	–	–	0.046	1.144	0.253
Educational level (associate degree or below/bachelor degree or above)	−0.063	−1.865	0.063	0.080	2.722	0.007	0.001	−0.008	0.994
Empathy training (yes/no)	−0.056	−1.686	0.093	0.033	1.156	0.248	0.001	0.011	0.991
Transition shock	−0.192	−4.647	0.000	0.430	11.915	0.000	0.514	10.574	0.000
Empathy	0.259	5.914	0.000	−0.189	−4.952	0.000	−0.100	−2.311	0.021
Resilience	0.343	6.947	0.000	−0.247	−5.732	0.000	−0.024	−0.411	0.681
Adaptive coping	0.107	2.492	0.013	−0.078	− 2.084	0.038	−0.047	− 0.933	0.351
Passive coping	−0.041	−1.098	0.273	0.098	2.999	0.003	0.149	3.393	0.001
	Adjusted R^2^ = 0.576, F = 77.196, *p* < 0.001			Adjusted R^2^ = 0.678, F = 118.741, *p* < 0.001			Adjusted R^2^ = 0.420, F = 36.445, *p* < 0.001		

*CS* compassion satisfaction, *STS* secondary traumatic stress

## Discussion

It was revealed that, higher transition shock contributed to less CS, and more burnout and STS, which indicated that NGNs who experienced more reality shock during the transition to clinical practice had lower CS and higher CF. The first year of clinical practice is an essential adjustment period for NGNs due to the lack of clinical competence [29]. The greatest reality-related shock NGNs perceived in the first year of job was the mis-match between actual and expected work and work environment [13]. Loss of social support, and incongruity in work and personal life were the greatest transition shock NGNs experienced in the first year of work [30]. Moreover, this study was conducted during the outbreak of the Covid-19 pandemic, in which in addition to shortages of personal protective equipment, inadequate staffing was one of the most pressing stressors experienced by nurses working in hospital settings, which negatively impacted nurse well-being [31]. Therefore, NGNs who enter nursing profession during the pandemic are likely to suffer from more reality shock in transition phase due to inadequate staffing and excessive workload. NGNs with more transition shock usually cannot effectively adapt to a real work environment and are susceptible to experience a variety of negative emotional states, less self-esteem and job involvement [32], which can result in increased CF and decreased CS. Accordingly, it is important to develop strategies (e.g., transition or preceptorship programmes) for facilitating the smooth transition and integration of NGNs, mitigating transition shock, hence promoting CS and reducing CF.

It was also revealed that, higher empathy significantly contributed to higher CS, and lower CF (burnout and STS) in NGNs, which are similar to the results of previous studies on experienced nurses [15, 16]. Empathy represents a caregiver’s ability to understand and share in clients’ thoughts and feelings from their perspectives, which can help identify clients’ needs, provide needs-based care, and facilitate clients’ satisfaction [33]. In turn, positive feedback from clients can assist in reducing nurse negative feelings driven by fear and work-related trauma, and enhancing the pleasure from helping others. On the other hand, empathic care can reflect nurses’ professional values, promote positive interactions between nurses and clients, and develop reciprocal trust within the therapeutic relationships, which can further facilitate CS and reduce CF in nurses including NGNs. Thus, based on our findings, it is essential to establish strategies of psychological formulation in increasing and sustaining empathy for NGNs.

Moreover, this study found that, resilience affected CS and burnout significantly, which are similar to the results of prior studies [19, 20]. Resilience refers to an adaptive process when encountering with stressful events and adversity. Individuals with higher resilience usually do not regard workplace stressors as a threat, and are more capable of adopting problem-solving coping, avoiding emotion-centered coping, and seeking social support to handle workplace stressors, which can result in positive compassionate feelings towards others and low perception of burnout [4]. However, this study did not support a significant effect of resilience on STS. The results are similar to the findings of a prior study on critical care nurses, which indicated that although resilience, as a predictor, significantly explained 15.4% of the variance of STS, its explaining effect on STS was lower than those on CS (66.0%) and burnout (22.0%) [20]. The results in our study suggest that, strategies for facilitating resilience training in NGNs are necessary in future studies to facilitate CS and alleviate burnout.

In parallel to the findings of prior studies [17, 21], it was revealed that, passive coping in NGNs was a significant contributor to CF, but it did not significantly predict CS. Coping is a process of fitting into the ward culture, and NGNs who adopt passive coping are susceptible to have low self-efficacy and be lack of confidence in dealing with stressful events, which may affect their ability to work with clients who suffer from stressful events and result in more CF. In this study, it was also revealed that, adaptive coping significantly contributed to CS, which is similar to the results of prior studies [21, 34]. Nurses with adaptive coping strategies usually search for social support and effective solutions when facing workplace stressors, and higher problem-solving coping significantly contributes to higher CS [21]. Therefore, it is pivotal to develop coping training programmes for NGNs, which focus on promoting adaptive coping and avoiding passive coping, in order to further enhance CS and reduce CF.

In addition, this study revealed that, NGNs with a bachelor degree or above reported higher burnout than those with an associate degree or below, but no significant differences on STS and CS were discovered between NGNs with different educational levels. The results are not similar to the findings of prior studies on experienced nurses, which indicated that educational level was not a significant predictor for each subscale of professional quality of life in cancer nurses or critical care nurses [17, 35]. Burnout refers to negative feelings of frustration and exhaustion due to difficulties in handling job-related stressors effectively [4]. In China, compared to NGNs with an associate degree or below, those with a bachelor degree or above usually receive more training on theoretical knowledge and critical thinking, and less training on clinical skills during school education, which may result in more frustration and hopelessness because of difficulties in coping with workplace stressors in the early phase of career development when clinical skills are highlighted.

### Limitations

Three limitations were noted. First, a cross-sectional design was used, which could not explore the causal mechanisms between the variables. Second, the samples were only selected from tertiary hospitals in Sichuan province, without samples from secondary and primary institutes, which might limit generalizability of the results. Finally, although the impact of transition shock, empathy, resilience and coping strategies on professional quality of life were examined, the reciprocal associations between the predictors (e.g., moderating or mediating effects) were not explored.

## Conclusion

This study indicates that, higher transition shock and lower empathy result in less CS and more burnout and STS. Higher adaptive coping and resilience cause higher CS and lower burnout. The results of our study offer a new insight for nursing managers in clinical settings to develop effective strategies to promote professional quality of life in NGNs. Strategies such as transition or preceptorship programmes, and empathy, resilience and coping training are effective methods to reduce transition shock, facilitate empathy, resilience and coping, and consequently, enhance professional quality of life in NGNs.

## Data Availability

The datasets generated and/or analysed during the present study are not publicly available due to the data being proprietary and confidential records of the West China Hospital/West China School of Nursing, Sichuan University, but are available from the corresponding author on reasonable request.

## Abbreviations

AC: Adaptive Coping
CS: Compassion Satisfaction
CF: Compassion Fatigue
NGN: Newly Graduated Nurses
PC: Passive Coping
STS: Secondary Traumatic Stress
TS: Transition Shock
